# The mediating role of ICT learning confidence and technostress between executive functions and digital skills

**DOI:** 10.1038/s41598-024-63120-w

**Published:** 2024-05-29

**Authors:** Cintia Bali, Zsuzsanna Feher, Nikolett Arato, Botond Laszlo Kiss, Beatrix Labadi, Andras Norbert Zsido

**Affiliations:** 1 Faculty of Humanities and Social Sciences, Institute of Psychology, University of Pécs, 6 Ifjusag Street, Pecs, 7624 Hungary; 2 Contemporary Challenges Research Centre, University of Pécs, Pecs, Hungary; 3 Institute of Psychology, Eotvos Lorand University, Budapest, Hungary; 4 Szentagothai Research Centre, University of Pécs, Pecs, Hungary

**Keywords:** Cognitive flexibility, Cognitive control, Digital skills, ICT attitude, Learning confidence, Technostress, Human behaviour, Quality of life

## Abstract

Having good digital skills is essential today, yet little is known about the cognitive factors that influence the development of these skills. Given the importance of executive functions (EFs) in adapting to environmental changes and acquiring skills, EFs might contribute to acquiring digital skills too. EFs might also influence people’s approach toward ICTs and affect digital skills through emotional variables. Therefore, here, we tested whether cognitive control and flexibility are connected to computer and smartphone skills through emotional factors (learning confidence, stress, and attitude). A total of 269 participants (56 males, *M* = 30 years) filled out our survey which included questionnaires on demographic variables (age, education, and socioeconomic status), ICT motivation, digital skills, EFs, and technology-related emotional factors. EFs were also investigated through two performance-based measures. We used generalized linear models and structural equation modeling to test the associations between these variables. The results showed that smartphone skills were positively associated with self-reported cognitive flexibility through ICT learning confidence and technology-induced stress. Self-reported cognitive control and age were connected to smartphone skills directly. Self-reported cognitive flexibility was also associated with computer skills through ICT learning confidence. In addition, performance-based cognitive control and the level of education were directly linked to computer skills. These results may provide guidance to support digital skills and create digital skill training.

## Introduction

Digital skills are becoming more and more important as digitalization is increasing in the field of work and education^1–3^. This is illustrated by the fact that individuals with better digital skills are more likely to find a job in the labor market^4^. Nowadays using ICTs is inevitable, yet 17% to 30% of people rate their digital skills lower than average according to recent studies^2,5^. This highlights that although ICT devices have become more available^6^, not everyone has the required level of digital skills^7,8^. To help individuals become more efficient users of modern technology; first, we need to identify the factors contributing to the acquisition of advanced digital skills. However, rather than focusing on the contributors of good digital skills, research tends to focus on ICT acceptance and adoption. This is reflected in the number of published articles on this subject. When referring to digital skills, we should distinguish between skills related to computers and smartphones. On the one hand, desktop computers, and notebooks have been part of our lives for a while, therefore we had time to get used to their presence in our lives and to use them. On the other hand, mobile devices, such as smartphones or tablets, are relatively new technologies which could make their acceptance harder, especially for older users^9^. However, they are also more user-friendly without all the peripherals and the necessity of learning to use and switch between them^10^. Taking all this into account we assume that the factors contributing to computer and smartphone skills might not be the same. It is, therefore, crucial to distinguish between computers and touchscreen devices (smartphones and tablets) when examining the determinants of strong digital skills.

The contribution of executive functions to learning digital skills is less researched than other factors, such as personality traits, attitudes, or sociodemographic variables^11,12^, despite their key role in adapting to unfamiliar situations and acquiring new skills^13^. Executive functions are essential for the acquisition of new skills and knowledge and, therefore likely to contribute to the development of digital skills. Executive functions are a set of top-down cognitive processes that support goal-directed behavior and foster flexible behavioral adaptation to unfamiliar circumstances^14^. These processes include various components such as working memory, inhibitory control, or cognitive flexibility^15–17^. Mitzner and colleagues^18^ found that individuals with better executive functions were more likely to use technology than their peers with poor performance. This might be because reduced working memory capacity (holding and manipulating information on our short-term storage^19^) and processing speed decrease performance on technology-based tasks^20^. Cognitive flexibility and cognitive control as components of executive functions^16,17^ could be particularly important in developing digital skills. Cognitive flexibility is the ability to shape our behavioral responses according to our continuously changing circumstances to solve problems and adapt to new environmental challenges^15,21^. Individuals with a higher level of cognitive flexibility are more prone to engage in cognitively challenging activities, learn new skills, or form a more positive attitude toward unfamiliar events or technologies^22,23^. This shows that cognitive flexibility can be a key factor in learning and mastering new technologies. On the other hand cognitive control allows us to maintain goal-directed behavior, inhibit distractors, and highlight the relevant environmental stimuli^15,24^. A high level of cognitive control enables effective learning by maintaining goal-directed behavior and focusing attention on the skill to be learned^25,26^, thus, presumably, it plays a role in getting familiar with current technologies and learning the correct ways to use them. Despite all this, the majority of the existing literature focuses on how technology contributes to healthy aging and the maintenance of cognitive functions^27,28^.

Although the relationship between technology use and cognitive functions is a widely researched topic, the contribution of cognitive functions to successfully acquiring digital skills is a neglected area. Recent studies focusing on people living with mild cognitive impairment (MCI) or dementia found that individual differences in cognitive functions (e.g., attention or visual memory) were key determinants in the use of digital technology^29,30^. More specifically people living with MCI faced more difficulties and required more support from their social environment in using ICT devices^30^. Research on patients living with MCI and dementia clearly shows that cognitive processes play a role in acquiring digital skills, however, the results say little about the specific mechanisms involved. Although these studies shed some light on the importance of cognitive functions in learning digital skills, they focused on people living with dementia and MCI, and they did not distinguish between smartphones and computers. Therefore, the impact of cognitive variables on digital skills in a healthy population is still unknown, and possible differences between skills related to smartphone and computer devices are yet unmapped.

Cognitive control and cognitive flexibility besides their direct impact can influence digital skills by forming emotional responses toward technology. A higher level of cognitive flexibility and cognitive control usually leads to more effective emotion regulation and resilient behavior^31,32^, which eventually will lead to better outcomes in learning situations^33,34^. ICT devices might raise anxiety or stress which can lead to avoidance and consequently less trained digital skills^35^. Stress induced by ICTs, termed as technostress^35^, is a widely investigated phenomenon especially since COVID-19 forced many individuals to incorporate a higher proportion of ICT use in their daily life^36,37^. It is therefore reasonable to assume that technological challenges may evoke negative feelings such as stress or anxiety. A recent neuroimaging study emphasized the importance of cognitive flexibility in the down-regulation of negative emotions through more effective cognitive reappraisal^38^. Cognitive reappraisal is also closely linked to cognitive control^39^, as is our ability to disengage our attention from negative emotional stimuli; the latter can help us not only focus on the downsides of a situation but notice the positives as well^40^. Cognitively flexible individuals with developed cognitive control functions are therefore more likely to focus on the opportunities offered by the technology (e.g., keeping in touch with loved ones) leading to more positive attitudes and a more open-minded approach toward technology. Overall, individuals with a higher level of cognitive control and flexibility might perceive ICT as less threatening and feel that they have more control over technology^41^. This reduces stress and anxiety induced by technological devices^42^. Consequently, these individuals will tend to demonstrate less avoidance and gain more experience with ICT devices^43^, which is essential for learning and developing new skills^44^. Above all, cognitive flexibility can also be a powerful component of digital skills because it is strongly related to self-confidence^45^. Therefore, cognitive flexibility through increased learning confidence might make individuals more ready to enter unfamiliar situations (e.g., using technology to work) and face uncertainty^46^. Consequently, individuals will have a chance to acquire related skills. Based on the relation of cognitive flexibility and cognitive control to emotion regulation, attitudes, and self-confidence it can be assumed that executive functions exert their effects on ICT skills not only directly, but through emotional factors.

Although adaptation to using ICTs is extensively researched^47,48^, recent studies are mostly interested in factors that affect willingness to use ICT. For instance, it has been shown that factors like age, education, socioeconomic status, and openness toward new experiences have a significant effect on ICT adoption behavior^10,49^. Yet, we still do not know whether acceptance is linked to actual skills. Also, the majority of studies focus on the elderly—mostly individuals above 65 years^10,50^, however, youngsters are also facing challenges because of the extensive digitalization of education and workplaces. This is confirmed by the fact that although students typically consider their digital skills to be good, in reality, their skills are rather average to low (at least one it comes to more complex and less tangible components of digital literacy)^51^. A deeper understanding of the factors influencing digital skills would help us to create an environment where these skills can be acquired. Further, more advanced digital skills could provide more benefits from using ICT. People can benefit not only in education and workplaces but also by making technology-based healthcare and mental health interventions more widely accessible.

**Figure 1:**
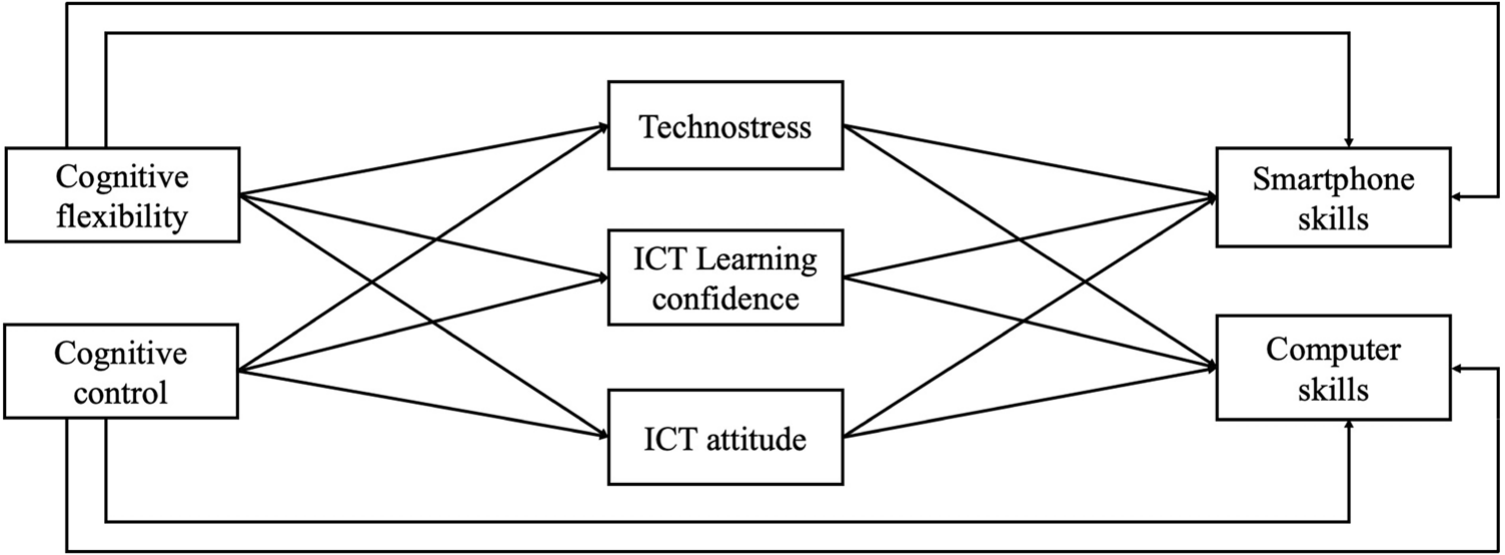
The proposed theoretical model regarding the mediating effect of technostress, ICT learning confidence, and ICT attitude between cognitive flexibility and cognitive control and digital skills (smartphone skills and computer skills).

In the present study, we aimed to explore the emotional and cognitive factors that contribute to the development of computer and smartphone skills. We predicted that cognitive flexibility and cognitive control via various factors (i.e., technostress, confidence, attitude toward ICT) are key features in the development of digital skills; thus, we proposed a model to test a combined effect of these factors on computer and smartphone skills (see Fig. 1). We hypothesized that cognitive flexibility and cognitive control would be positively associated with smartphone and computer device skills by down-regulating negative thoughts and emotions associated with technology, contributing to a more positive attitude and increasing the confidence to learn about technology.

## Method

### Sample

We recruited 269 participants (56 males, 2 preferred not to answer), aged between 18 and 74 years (*M* = 30 years, *SD* = 14.3 years) through social media and mailing lists. The majority of the participants are currently studying at university and consider their monthly income to be in line with the Hungarian average. On average participants own at least 2 devices and spend at least 6 h using some form of ICT for work, study, or communication. The demographic data are presented in Table 1, while means and standard deviations for the questionnaires and behavioral measures are presented in Table 2.

**Table 1:** Descriptive data of the sample regarding education (highest level of education of the participants), socioeconomic status (SES) (numbers and percentages), screen time (for work, study, and communication), the number of owned devices, and ICT motivation (means and standard deviations).

	N		%			
Highest level of education						
Elementary school	3		1			
Technical school	1		0.4			
High school	42		15.5			
Currently studying in higher education	125		46.5			
College/University	98		36.5			
Socioeconomic status (monthly income)						
Below 50.000 HUF	18		7			
50.000–100.000 HUF	40		15			
100.000–200.000 HUF	101		37.5			
200.000–300.000 HUF	68		25			
300.000–400.000 HUF	23		8.5			
Above 400.000 HUF	19		7			

Screen time	Work		Study		Communication	
	N	%	N	%	N	%
Not at all	105	39	60	22.3	2	0.7
Less than 30 min	9	3.3	13	4.8	34	12.6
30 min	7	2.6	14	5.2	53	19.7
1 h	19	7.1	26	9.7	81	30.1
2 h	16	5.9	49	18.2	56	20.8
3 h	21	7.8	40	14.9	18	6.7
4 h or more	92	34.2	67	24.9	25	9.3

	M		SD			
The number of devices	2.48		0.826			
ICT motivation	13.88		2.689

**Table 2:** Mean scores (M) and standard deviations (SD) of Cognitive Flexibility Scale (self-reported cognitive flexibility), Inattention and Hyperactivity-Impulsivity (self-reported cognitive control), perseverative responses on Wisconsin Card Sorting Test (performance-based cognitive flexibility), the Flanker effect in milliseconds (performance-based cognitive control), Technostress Scale, ICT Learning Confidence Scale, Internet Attitude Scale, Mobile Device Proficiency Questionnaire (smartphone skills) and Computer Proficiency Questionnaire (computer skills).

		M	SD	Min	Max
Self-reported cognitive flexibility		52.03	7.93	30	72
Self-reported cognitive control	Inattention	14.58	5.99	2	34
	Hyperactivity-Impulsivity	14.38	5.33	0	32
Performance-based cognitive flexibility		7.72	3.54	5	22
Performance-based cognitive control		25.2	29.2	− 81.6	118
Technostress		35.15	8.84	14	57
ICT Learning Confidence		18.63	5.46	9	38
ICT Attitude		61.82	6.54	42	72
Smartphone skills		70.48	9.24	15	75
Computer skills		55.88	4.58	37	60

Participation was voluntary. The required sample size for this experiment was determined by computing estimated statistical power (*RMSEA* = 0.05, *β* > 0.8, *alpha* = 0.05) using the SEM Power package for R^52,53^. The analysis indicated a required total sample size of 241; thus, our study was adequately powered.

The study was approved by the Hungarian United Ethical Review Committee for Research in Psychology (reference nr. 2022–98) and was carried out following the Declaration of Helsinki. Informed written and verbal consent was obtained from all participants. At the beginning of the questionnaire, participants declared that they had no psychiatric disorder.

### Materials

#### Sociodemographic questions and ICT motivation

Participants filled out questions regarding their age, gender, the highest level of education, and objective socioeconomic status (SES). SES was measured by the monthly income of the participants given in Hungarian forint. Additionally, we asked them about the number of ICT devices they own and their amount of screen time on an average day. For the latter participants rated three items (education, work, communication) on a 7-point Likert-type Scale (1—None 7—more than 4 h). With four items we also assessed how motivated they felt to use ICT devices. Participants rated the level of their motivation toward using ICT on a 5-point Likert-type scale (1—Not at all to 5—Very much). Based on prior studies^54,55^ we considered the following aspects of using ICT: hedonic, communicational, informational, and self-featuring purposes. For the detailed descriptive data of the sample see Table 1.

#### Computer and smartphone skills

To measure computer skills in various sets of computer-related activities we used the 12-item short version of the Computer Proficiency Questionnaire (CPQ)^56^. Participants had to rate each item on a 5-point Likert-type scale (1—Never tried to 5—Very easily) according to which number best describes the level of their abilities in the given computer-related activity (e.g., *‘I can use a mouse’, ‘I can load ink into the printer’*). Higher scores suggested more advanced computer skills. In this study, the McDonald’s omega was 0.88.

To assess how participants rate their skill set in using smartphones we used the 16-item short version of the Mobile Device Proficiency Questionnaire (MDPQ)^9^. Participants were asked to rate each item (e.g*., ‘I can use the onscreen keyboard to type’, ‘I can set up a password to lock/unlock the device’*) on a 5-point Likert-type scale (1—Never tried to 5—Very easily) according to which number best describes the level of their abilities in the given touchscreen device-related activity. Higher scores meant more advanced skills in using touchscreen devices. In both questionnaires the participants had to rate the ease of use of each function featured in the items. In this study, the McDonald’s omega was 0.95.

#### Technostress

We used the 16-item Technostress Scale (TS) to measure the subjective level of ICT-induced stress^35^. Items (e.g., *‘The constant developments and upgrades in the technology are a burden for me’*) are rated on a 5-point Likert-type Scale (1—Strongly Disagree to 5—Strongly Agree). Higher scores indicated a higher level of stress induced by technology. In this study, the McDonald’s omega was 0.79.

#### ICT learning confidence

We used the ICT Learning Confidence Scale (ILCS), the modified version of the Computer Anxiety Rating Scale (CARS) to measure ICT learning confidence^57^. The original scale was developed to measure computer anxiety, however, to match the aim of our study we changed the term ‘computer’ to ‘ICT devices’. CARS consists of five subscales and 19 items, however, we only used three subscales (9 items) to measure the willingness to use ICT, i.e., the Appeal of learning about and using computers (*‘If given the opportunity, I would like to learn about and use ICT devices.’—the item is reverse coded*), Learning computer skills (*‘I am confident that I can learn computer skills.’—the item is reverse coded*), and Traits to overcome anxiety (*‘You must be a genius to understand all the special keys on the keyboard.*’). We included the items from these factors because they correspond well with learning confidence in a technological context. For the items see Supplementary Material 1. Participants were asked to rate each item on a 5-point Likert-type scale (1—Strongly Disagree to 5—Strongly Agree). All but two items are reverse coded, thus lower values on the scale indicated greater confidence in ICT learning and lower levels of avoidance. The McDonald’s omega was 0.79.

#### ICT Attitude

To measure attitudes toward ICT devices, we used a modified version of the Internet Attitude Scale (IAS)^58^. Originally this scale was developed for assessing high schoolers’ attitudes toward the Internet. For the present study, we changed the term ‘internet’ to ‘ICT devices’ and transformed the school-related items to make them relevant to everyday life (e.g., “*I only use the Internet at schools when told to*” was transformed to “*I only use ICT when told to*”). The scale consists of 18 items; participants were asked to rate the items on a 4-point Likert-type scale (1—Strongly Disagree to 4—Strongly Agree). Higher scores indicated a more positive attitude toward ICT devices in general. In the present study, the McDonald’s omega was 0.86.

#### Cognitive flexibility

We used the Wisconsin Card Sorting Test (WCST) as a performance-based assessment tool to test cognitive flexibility at a behavioral level^59^. The 64-card version contains 4 stimulus cards and 64 response cards which are varied in three dimensions, i.e., color, shape, and number. Participants were instructed to match the response cards appearing on the left with one of the stimulus cards (4 per trial) presented in the upper section of the screen. They could choose the right match based on one of the following criteria: color, shape, or number of the figures presented on the cards. Participants received feedback after each choice showing if their response was correct or not. Participants had to figure out the current sorting criterion based on the feedback in a trial–error method. The sorting criterion changed after every 10th response. When a response matched the previous sorting criterion instead of the current sorting criterion, it was considered a perseverative response^60^. The total number of perseverative responses was used as an indicator of cognitive flexibility.

Additionally, we used the Cognitive Flexibility Scale (CFS)^61^ to measure the self-reported level of the ability to adapt to new events and changing circumstances^21^. CFS is a one-factor scale containing 12 items. Participants rated each item on a 6-point Likert-type scale (1—Strongly Disagree to 6—Strongly Agree). Higher scores indicated a higher level of cognitive flexibility and better adaptability. In this study, the McDonald’s omega was 0.84.

#### Cognitive control

Flanker paradigm^62^ was used as a performance-based assessment of executive control. During the task combinations of five letters (e.g., XXCXX) appeared in the center of the screen. Participants were asked to indicate which letter was in the middle (i.e., the target). If the target was ‘X’ or ‘C’ they had to press the button ‘A’ if the target was ‘V’ or ‘B’ they were asked to press ‘L’ on the keyboard. Half of the trials were congruent, where the response to the target and the distractors were the same (e.g., XXXXX or XXCXX). The other half were incongruent trials, where the response to the target and the distractors differed (e.g., XXVXX or VVXVV). Each participant completed a total of 150 trials in random order. Participants had up to 2000 ms to respond. Before the task participants practiced the task with a total of 16 trials to get familiar with the paradigm and learn the responses on the keyboard.

Additionally, the World Health Organization adult ADHD (attention-deficit/hyperactivity disorder) self-report scale (ASRS) was used to measure inattention, and hyperactivity-impulsivity^63^. The 18-item questionnaire has two subscales i.e., Inattention and Hyperactivity-Impulsivity. Items were rated on a 5-point Likert-type scale (1—Never to 5—Very Often) according to which number best describes the behavior of the participants in the past six months. Higher scores indicated a higher level of inattention and more frequent hyperactive and impulsive behavior. In this study, the McDonald’s omegas were 0.83 for Inattention and 0.72 for Hyperactivity-Impulsivity.

### Procedure

Participants were asked to fill out the questionnaires, and then complete the two performance-based tasks through Psytoolkit^64^, a free software for running psychological experiments online. The survey was distributed through social media sites and email lists, and it was only available on computer devices. Participation was voluntary, and informed consent was obtained from all the participants. Participants were informed that they could withdraw from the study at any point without consequences. Participants did not receive compensation for their participation.

### Statistical analyses

First, we carried out four separate Generalized Linear Models (GLM) to explore the relationship between the variables that comprise our theoretical model. In our final model, we included variables based on their significance in the GLMs for Structural Equation Modeling. This was necessary to reduce the complexity of our original model, making it easier to understand and generalize. Regarding computer skills, smartphone skills, and performance-based cognitive flexibility, the data were not normally distributed. Apart from these two, the distribution of all variables was normally distributed as the absolute values of Skewness and Kurtosis were below 2.

To explore the significant contributors of computer and smartphone skills we performed two separate GLMs with computer skills and smartphone skills as outcome variables. Affective (technostress, ICT learning confidence, ICT attitude) and cognitive (self-reported cognitive flexibility, performance-based cognitive flexibility, performance-based cognitive control, and self-reported cognitive control which consisted of inattention and hyperactivity-impulsivity) variables were included as independent predictors. For the performance-based cognitive control, we used the Flanker effect scores, which is the difference between the mean RTs of the correct responses on incongruent and congruent trials. This value is considered as an indicator of executive control. Subsequently, we performed two GLMs to test the cognitive variables’ predictive value on the affective variables. Technostress and ICT learning confidence scores were entered as outcome variables (again, in two separate models) and cognitive variables (same as previously) were included as independent predictors. We did not perform a GLM with the ICT attitude scores as an outcome variable because it was not associated with computer skills and smartphone skills. Based on theoretical considerations we controlled for the sociodemographic variables (age, education, SES, screen time, number of devices) and ICT motivation in all four GLMs. GLM analyses were performed using the Jamovi statistical software version 2.3^65^.

**Figure 2:**
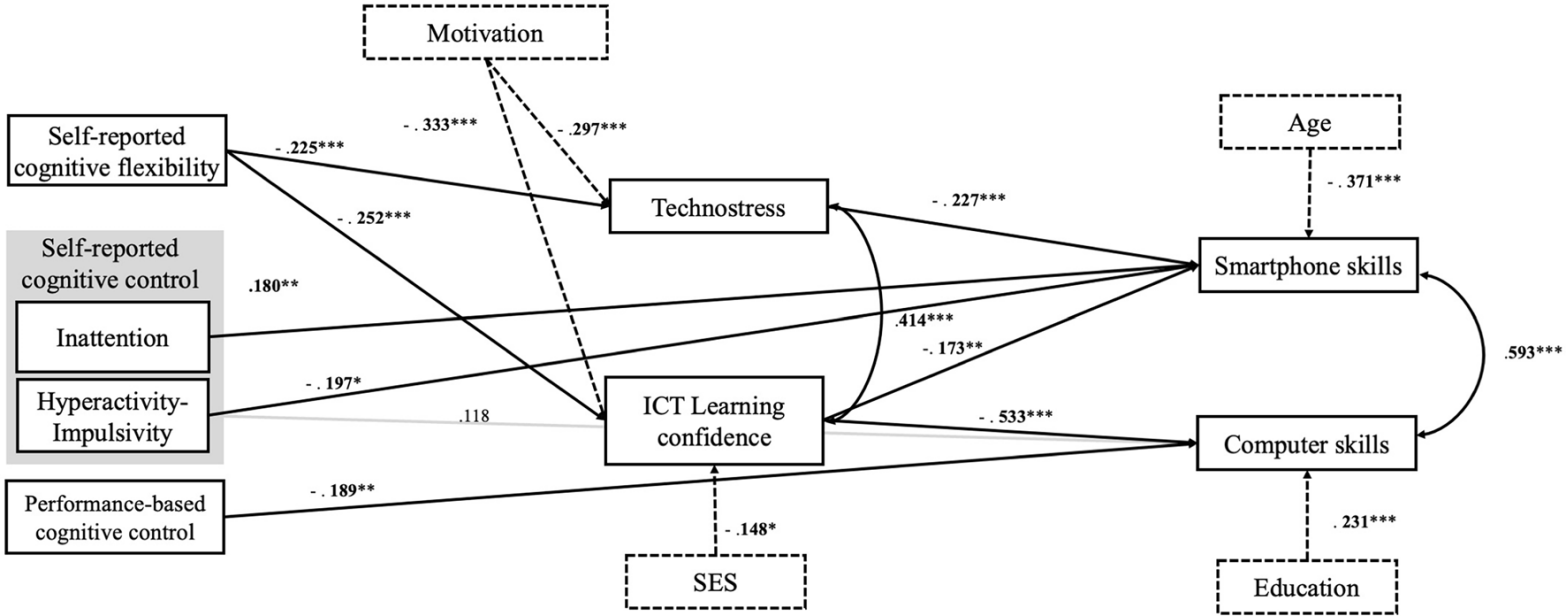
The model we tested on the potential contributors of digital skills based on the GLMs. All pathways are displayed. Statistically significant pathways are highlighted in black (* p < 0.05, ** p < 0.01, *** p < 0.001). All reported estimates are standardized point estimates. Grey lines indicate nonsignificant pathways. Control variables are presented with dashed lines. Self-reported cognitive flexibility = Cognitive Flexibility Scale, Self-reported cognitive control = Hyperactivity-Impulsivity and Inattention subscales, Performance-based cognitive control = Flanker effect, Technostress = Technostress Scale, ICT Learning confidence = ICT Learning Confidence Scale, Smartphone skills = Mobile Device Proficiency Questionnaire, Computer skills = Computer Proficiency Questionnaire, SES = Socioeconomic status. Education = Highest level of education.

Then, we performed Structural Equation Modelling (SEM) to assess fit measures for our proposed model (see Fig. 2) which we based on the results of the previous GLM analyses. To ensure that the distribution of variables does not bias the results, we standardized the variables before entering them into the model and used a robust estimator in the analysis. Achieved scores on computer skills and mobile skills were entered into the model as outcome variables, while ICT learning confidence, technostress, self-reported cognitive flexibility, inattention, hyperactivity-impulsivity, and performance-based cognitive flexibility were included as predictor variables. After theoretical consideration, we allowed covariations between technostress and ICT learning confidence scores, and mobile skills and computer skills scores. The model was controlled for sociodemographic factors (age, SES, highest levels of education, screen time), and ICT motivation. All variables were entered as measured variables.

For the SEM analysis, we used the diagonally weighted least squares (DWLS) estimator^66^. To evaluate the model fit, we used the relative chi-square (χ^2^/df), comparative fit index (CFI), Tucker–Lewis index (TLI), root mean square error of approximation (RMSEA), and the standardized root mean squared residual index (SRMR). The cut-offs for good model fit were a relative chi-square of 3 or lower, CFI and TFI values of 0.95 or greater^67^, and RMSEA and SRMR values of 0.08 or lower^68^. The SEM model was tested using the JASP statistical software version 0.16.3 for Windows^69^ utilizing the lavaan package for R^70^.

## Results

### Generalized linear models (GLM)

#### Associations of digital skills with cognitive and affective variables

To analyze the associations of digital skills with affective and cognitive variables we performed two GLMs separately for smartphone and computer skills. See Table 3 for statistical results including ORs, p-, and χ^2^ values. The analyses showed that computer skills were associated with hyperactivity-impulsivity, performance-based cognitive flexibility, ICT learning confidence, and the level of education of the participants. Smartphone skills were associated with inattention, hyperactivity-impulsivity, technostress, and ICT learning confidence scores, and the age of the participants. There were no other observed significant associations between the variables.

**Table 3:** *GLM 1&2:* The associations of digital skills with cognitive factors, affective factors, and sociodemographic variables.

GLM 1 & 2		Computer skills		Smartphone skills	
		OR	p	OR	p
Self-reported cognitive flexibility		1.075	0.101	0.946	0.413
Self-reported cognitive control	Inattention	1.033	0.560	*1.288*	*0.023*
	Hyperactivity-Impulsivity	*0.860*	*0.020*	*0.717*	*0.010*
Performance-based cognitive control		*0.970*	< *0.001*	0.971	0.106
Performance-based cognitive felxibility		0.984	0.819	0.980	0.885
Technostress		0.973	0.431	*0.803*	*0.001*
ICT Learning confidence		*0.756*	< *0.001*	*0.745*	*0.009*
ICT Attitude		0.976	0.546	0.885	0.131
Age		1.016	0.474	*0.798*	< *0.001*
Education		*3.270*	*0.002*	1.266	0.748
SES		0.913	0.673	1.030	0.945
Screen media		1.022	0.799	0.999	0.994
Device		1.305	0.398	3.127	0.069
ICT motivation		1.253	0.051	1.306	0.242
χ^2^		1694.39***		7830.89***	

ORs, p-values, and χ^2^ values (* p < .05, ** p < .01, *** p < .001) are presented. Significant associations are italicized. Self-reported cognitive flexibility = Cognitive Flexibility Scale, Self-reported cognitive control = Hyperactivity-Impulsivity and Inattention subscales, Performance-based cognitive control = Flanker effect, Technostress = Technostress Scale, ICT Learning confidence = ICT Learning Confidence Scale, Smartphone skills = Mobile Device Proficiency Questionnaire, Computer skills = Computer Proficiency Questionnaire, SES = Socioeconomic status, Device = Number of the owned ICT devices.

#### Associations of the affective factors with cognitive variables

To explore the associations of the affective variables with the cognitive variables we performed two separate GLMs. See Table 4 for statistical results including ORs, p-, and χ^2^ values. The analyses showed that technostress scores were associated with self-reported cognitive flexibility scores and ICT motivation scores, while ICT learning confidence scores were associated with self-reported cognitive flexibility scores, ICT motivation scores, and the socioeconomic status of the participants. There were no other observed significant associations. We did not perform a GLM with ICT attitude scores as a dependent variable, since ICT attitude scores had no significant associations with computer and smartphone skills in the preceded GLMs. For all statistical values including point estimates, standard errors (SE), ORs (exp(ß)), and their 95% CI, z values, and* p *values of the GLMs see Supplementary Table 1.

**Table 4:** *GLM 3&4:* The associations of the affective variables, cognitive variables, and sociodemographic variables.

GLM 3 & 4		Technostress		ICT Learning confidence	
		OR	p	OR	p
Self-reported cognitive flexibility		*0.782*	< 0*.001*	*0.893*	*0.007*
Self-reported cognitive control	Inattention	0.972	0.802	0.940	0.370
	Hyperactivity-Impulsivity	1.190	0.189	1.064	0.436
Performance-based cognitive control		0.986	0.437	1.018	0.119
Performance-based cognitive flexibility		0.947	0.718	1.020	0.825
Age		1.050	0.263	0.960	0.125
Education		0.878	0.864	0.594	0.258
SES		0.517	0.126	*0.599*	*0.050*
Screen media		1.055	0.748	0.893	0.266
Device		0.431	0.192	0.786	0.538
ICT motivation		*0.453*	< 0*.001*	*0.416*	< 0*.001*
χ^2^		3430.87***		1824.28***	

ORs, p-values, and χ^2^ values (* p < 0.05, ** p < 0.01, *** p < 0.001) are presented. Significant associations are italicized. Self-reported cognitive flexibility = Cognitive Flexibility Scale, Self-reported cognitive control = Hyperactivity-Impulsivity subscale and Inattention subscale, Performance-based cognitive control = Flanker effect, Technostress = Technostress Scale, ICT learning confidence = ICT Learning Confidence Scale, Smartphone skills = Mobile Device Proficiency Questionnaire, Computer skills = Computer Proficiency Questionnaire, SES = Socioeconomic status, Device = Number of the owned ICT devices.

### Structural equation modelling

We used SEM to test the direct and indirect pathways between the cognitive variables, affective variables, and digital skills. Only the significant associations in the GLMs were included in the model. The test yielded a good model fit (χ^2^/*df* = 1.15, *p* = 0.278*,* CFI = 0.991, TLI = 0.985, RMSEA = 0.025, 90% CI = [0.000–0.060], SRMR = 0.032). Smartphone skills (*R*^*2*^ = 0.312) were associated *negatively* with ICT learning confidence scores, technostress scores, hyperactivity-impulsivity scores, and *positively* with inattention scores. Computer skills (*R*^*2*^ = 0.362) were associated negatively with ICT learning confidence scores and performance-based cognitive control. Technostress scores (*R*^*2*^ = 0.154) and ICT learning confidence scores (*R*^*2*^ = 0.221) were associated with self-reported cognitive flexibility scores negatively. Hyperactivity-impulsivity scores were not associated with computer skills. Additionally, technostress scores were associated negatively with ICT motivation scores, while ICT learning confidence scores were associated negatively with both ICT motivation scores and SES. The age of the participants was negatively associated with smartphone skills, and the levels of education were associated with computer skills. For the exact ß values see Fig. 2 and for all statistical values including the point estimates, standard errors (SE), ß values, z values, and* p *values for both direct and indirect pathways see Supplementary Table 2.

Regarding the indirect pathways, we found that self-reported cognitive flexibility was associated with smartphone skills through technostress (ß = 0.051, *p* = 0.012) and ICT learning confidence scores (ß = 0.044, *p* = 0.013). Additionally, self-reported cognitive flexibility scores were associated with computer skills through ICT learning confidence (ß = 0.134, *p* < 0.001). Further, regarding covariances, both smartphone skills and computer skills and technostress and ICT learning confidence scores were positively associated. See Statistical results are reported in Fig. 2 for the model, and Supplementary Table 3 to see correlational coefficients across all included variables.

## Discussion

ICT devices have the potential to improve the quality of life and provide benefits in work, education, and healthcare^28,71,72^. Hence, individuals refusing to use ICT or lacking crucial digital skills are missing out on these advantages^9^. Identifying the contributing factors in the development of digital skills is crucial to helping people develop digital skills and benefit from using ICT devices. Therefore, our study aimed to explore the relationship between cognitive flexibility, cognitive control, technostress, ICT learning confidence, ICT attitude, and digital skills. We hypothesized that better cognitive functions would predict higher smartphone and computer skills through down-regulating technostress and increasing ICT learning confidence and positive attitudes toward the use of ICT. Our results, in general, supported this hypothetical model. However, the ICT attitude was omitted from the final model, as it was not related to either cognitive variables or digital skills in the preliminary analyses. This suggests that one’s general beliefs and evaluations of ICT devices do not influence the possession of digital skills. This is supported by the fact that although participants generally rated their ICT skills as high, there was still a wide variation in their attitudes towards these devices. According to a recent study^73^ attitudes do not always predict one’s actual intentions or behavior as their predictive value depends on many factors such as the strength or stability of the attitudes, and earlier experiences with the object^74^. Consequently, more positive attitudes toward ICT devices not necessarily be reflected in a higher technological commitment (e.g., learning about technology or more patience toward technology) and more advanced digital skills or vice versa. This also highlights that technology acceptance does not necessarily lead to more advanced digital skills, i.e. further research should focus on actual skills alongside technology acceptance. First, based on the significant associations in our final model we will discuss the determinants of computer skills, and the discussion of the contributors of smartphone skills will follow this. Finally, we address the sociodemographic variables and motivational factors.

A higher level of self-reported cognitive flexibility was associated with better computer skills via ICT learning confidence. This suggests that individuals who perceive themselves as cognitively flexible are more confident in learning about technology and are more ready to use ICT devices. In general cognitive flexibility allows more confidence in our abilities, which makes it possible to face challenging tasks^45^. This seems to be applied to the technological environment as well. Consequently, the confidence in engaging with technology will lead to more advanced computer skills presumably through more widespread use^44^ and persistent learning approach^75^. It is important to note though, that only the self-report assessment of cognitive flexibility was a significant predictor, while the performance-based measure was not. This discrepancy might indicate that computer skills are more affected by how flexible people see themselves rather than actual neuro-cognitive background mechanisms. We also found that individuals with better cognitive control, as indicated by the behavioral measure, had more advanced computer skills. Individuals with better cognitive control functions might be more susceptible to learning new technologies, spend more time exploring them in-depth, and might be less frustrated by the inconveniences caused by technology^29,30^. It shall be noted that this was only evidenced by the behavioral but not the self-reported measure. This discrepancy could be explained by the fact that individuals tend to misjudge their cognitive control capabilities^76^, leading to less accurate measurement in the case of a self-reported questionnaire. That is, the underlying neuro-cognitive mechanisms of executive control are better predictors of computer skills, regardless of how people perceive themselves in this matter. Furthermore, it is important to note that ASRS was designed to assess a wide range of ADHD-related symptoms based on DSM-IV^63^, not only cognitive control. Therefore, our result suggests that computer use-related skills are more influenced by cognitive control alone, than the constructs of inattention, hyperactivity, and impulsivity.

Smartphone skills were predicted by self-reported cognitive flexibility through technostress and ICT learning confidence, and, also by inattention and hyperactivity/impulsivity. These results indicate that individuals with a lower level of self-reported cognitive flexibility experienced a higher level of technostress and, at the same time, learning and using smartphones seemed less appealing to them. That is, individuals who consider themselves more adaptable might perceive relatively new technologies (such as smartphones or tablets) as less threatening and more controllable^15,21,41^. Reduced stress levels besides decreasing avoidance toward smartphones can also contribute to more effective learning and memory encoding^77^. Our results suggest that improving self-perceived cognitive flexibility would help reduce technological stress. This is further supported by that cognitive flexibility improves cognitive restructuring skills^78^ and a new positive perspective toward smartphones may increase the behavioral intention of learning about them. This can lead to more advanced smartphone skills. Technostress was predicted by self-reported cognitive flexibility suggesting that individuals considering themselves less adaptable and self-efficient perceive technology as more threatening and they presumably cope worse with excessive digitalization. This confirms our previous assumption that people’s preconceptions about themselves might be a better indicator of technology-related emotions than the actual neurological mechanisms underlying cognitive flexibility. Technostress was a significant contributor to smartphone skills, but not to computer skills. This might be because IT classes in school^79,80^ and the increasing digitalization of workplaces and education^1,4^ force individuals to use computers and learn the basics regardless of the technostress they experience. Beyond cognitive flexibility, self-reported cognitive control was directly associated with smartphone skills. Interestingly, individuals considering themselves inattentive acquired more advanced skills, while those with a higher level of hyperactivity and impulsivity reported less developed smartphone expertise. Previous studies have shown that the risk of problematic smartphone use or smartphone addiction was increased among people with ADHD and particularly those with inattention symptoms^81,82^. The increased screen time could result in better skills^83^ through excessive experiences, however, this correlation is not always evident^51^. In contrast, hyperactive and impulsive traits (even without a diagnosis) may inhibit deeper understanding^84^, leading to only superficial knowledge of modern technology, despite its intense usage.

Regarding the sociodemographic variables age, highest level of education, and socioeconomic status were found as significant contributors to digital skills or affections toward technology. Younger users had more advanced skills, while older individuals had less developed smartphone skills, which underscores the need for educating the elderly on smartphone usage. The significance of education and training on technology use is also supported by the fact that those who saw the benefits (e.g., keeping in touch, getting information) of technology and were more motivated to use it reported lower technostress and higher ICT learning confidence. In addition, individuals with a higher level of education reported better computer skills. Higher socioeconomic status was also associated with more learning confidence and less avoidance of technology, which was further facilitated by motivational factors. We primarily focused on the main motivational factors such as keeping in touch, getting information, self-promotion, or entertaining. All these results are in line with previous studies^10,49^, saying that besides executive functions socioeconomic status and ICT motivation have a crucial role in forming emotional responses toward technology. Digital skill training should focus on the obtainable benefits to increase motivation and reduce negative emotions toward ICT devices.

Some limitations of the present study shall be noted. We mostly used self-reported questionnaires which may have biased our results. Although we also included performance-based tests to measure cognitive flexibility and cognitive control, data were collected online, therefore environmental distractors were not controlled. This could have affected participants' performance on the performance-based tests and reduced the reliability of the results. It has also been shown previously that individuals often overestimate their digital skills^51^. However, in the mentioned study, less tangible components of digital literacy were studied (e.g. privacy), whereas in the current study, we asked about specific activities (e.g. opening emails, using an onscreen keyboard, etc.). It can be assumed that for the latter, individuals have more accurate knowledge and feedback about their skills. Thus, their judgments may be more accurate, but the possibility that the digital skill questionnaires are biased toward higher scores cannot be excluded. Collecting data online had another drawback; participants must have had at least basic levels of practice in using ICT devices. Our survey, therefore, did not have the potential to reach those who are not able to or not motivated to use ICT devices. This is clear from the questionnaire data, which suggests that our sample has good digital skills. Although this may limit the generalizability of the results, there was a wide variation in participants' attitudes towards ICT devices, meaning that attitudes and skills do not always go hand in hand. In the future, it would be crucial to use tests and obtain data in person to assess a more representative sample and get more accurate results. Furthermore, personal data collection would allow the inclusion of observational data in addition to self-reported data when assessing digital skills. The effect of education and sociodemographic variables on using ICT is well established in the literature^10,49,50^ therefore it is crucial to reach a wider range of respondents in the future. This could be also achieved through face-to-face data collection. Further, although we used structural equational modeling, this is only a cross-sectional study, which does not allow us to make causal conclusions. A longitudinal study would provide more exact results and would help us to determine the direction of the relationship between the measured variables.

A deeper understanding of the cognitive factors contributing to digital skills can provide us guidance to support individuals to learn advanced skills and benefit more from using ICT devices. There are proven benefits of using ICT^47,71^, yet many people still lack digital skills^5,8^. Our results emphasize the importance of self-reported cognitive flexibility, cognitive control, learning confidence, and stress induced by technology in acquiring smartphone and computer skills. These outcomes could support ICT adaptation and mastering digital skills. Also, these results could provide foundations for digital skill training. It seems that it may be worth focusing on one’s beliefs about one’s cognitive flexibility. A high level of self-reported cognitive flexibility could help acquire advanced digital skills by embracing the belief that anyone can learn to use technology. It can also reduce technology-related stress and increase learning confidence. Besides the importance of cognitive flexibility, our results suggest that individuals with a lower level of cognitive control tend to have less developed computer skills. Therefore, when designing training we must pay particular attention to the fact that people with weaker executive control might be more impeded in acquiring computer skills. As further contributors, we should also consider sociodemographic factors such as the highest level of education, socioeconomic status, and age as well as motivational factors.

### Supplementary Information

Supplementary Information 1.

Supplementary Table 1.

Supplementary Table 2.

Supplementary Table 3.

## Data Availability

The data set that includes computed study variables is available on the Open Science Framework: https://osf.io/zxag5/?view_only=fa16d86c5af64f73af1c3b7b3b498928.
