# Supplementary material for: The mediating role of ICT learning confidence and technostress between executive functions and digital skills
Source: Sci Rep. 2024 May 29;14:12343. doi: 10.1038/s41598-024-63120-w (PMC11136953; doi:10.1038/s41598-024-63120-w)
Supplement: Supplementary file 1 — Supplementary Information 1. [file 41598_2024_63120_MOESM1_ESM.docx]

**Supplementary material 1**

**ICT Learning Confidence Scale**

The challenge of learning about ICT is exciting.

If given the opportunity, I would like to learn about and use ICT devices.

I look forward to using ICT devices on my job.

I feel ICT devices are necessary tools in both educational and work settings.

Learning to operate ICT devices is like learning any new skill - the more you practice, the better you become.

I am confident that I can learn digital skills.

I do not think I would be able to learn a computer programming language.

You must be a genius to understand all the special keys contained on the keyboard.

Anyone can learn to use ICT devices if they are patient and motivated.
