# Supplementary material for: The mediating role of ICT learning confidence and technostress between executive functions and digital skills
Source: Sci Rep. 2024 May 29;14:12343. doi: 10.1038/s41598-024-63120-w (PMC11136953; doi:10.1038/s41598-024-63120-w)
Supplement: Supplementary file 2 — Supplementary Table 1. [file 41598_2024_63120_MOESM2_ESM.docx]

| Supplementary Table 1 - GLM 1&2: The associations of digital skills with cognitive factors, affective factors, and sociodemographic variables. GLM 3&4: The associations of the affective variables, cognitive variables, and sociodemographic variables. All statistical values are reported. | | | | | | | |
| --- | --- | --- | --- | --- | --- | --- | --- |
|  |  |  |  | **95% Exp(B) Confidence Interval** | |  |  |
| **Independent predictors** | **Estimate** | **SE** | **exp(B)** | **Lower** | **Upper** | **z** | **p** |
| **GLM1 - Computer skills** |  |  |  |  |  |  |  |
| Self-reported cognitive flexibility | 0.05423 | 0.0348 | 1.056 | 0.986 | 1.130 | 1.5584 | 0.120 |
| Inattention | 0.03683 | 0.0568 | 1.038 | 0.928 | 1.160 | 0.6484 | 0.517 |
| Hyperactivity-Impulsivity | -0.14009 | 0.0659 | 0.869 | 0.764 | 0.989 | -2.1274 | 0.034 |
| Performance-based cognitive flexibility | 0.01286 | 0.0729 | 1.013 | 0.878 | 1.169 | 0.1763 | 0.860 |
| Technostress | -0.01538 | 0.0348 | 0.985 | 0.920 | 1.054 | -0.4422 | 0.659 |
| ICT learning confidence | -0.30471 | 0.0572 | 0.737 | 0.659 | 0.825 | -5.3318 | < .001 |
| ICT attitude | -0.03213 | 0.0415 | 0.968 | 0.893 | 1.050 | -0.7750 | 0.439 |
| Age | 0.00789 | 0.0218 | 1.008 | 0.966 | 1.052 | 0.3619 | 0.718 |
| Education | 1.07298 | 0.3764 | 2.924 | 1.398 | 6.115 | 2.8503 | 0.005 |
| SES | -0.14252 | 0.2193 | 0.867 | 0.564 | 1.333 | -0.6498 | 0.516 |
| Screen time | 0.00130 | 0.0853 | 1.001 | 0.847 | 1.184 | 0.0152 | 0.988 |
| Device | 0.18528 | 0.3201 | 1.204 | 0.643 | 2.254 | 0.5789 | 0.563 |
| Motivation | 0.16237 | 0.1158 | 1.176 | 0.937 | 1.476 | 1.4025 | 0.162 |
| **GLM2 - Smartphone skills** |  |  |  |  |  |  |  |
| Self-reported cognitive flexibility | -0.05721 | 0.0678 | 0.944 | 0.827 | 1.079 | -0.8434 | 0.400 |
| Inattention | 0.25717 | 0.1107 | 1.293 | 1.041 | 1.607 | 2.3230 | 0.021 |
| Hyperactivity-Impulsivity | -0.32269 | 0.1283 | 0.724 | 0.563 | 0.931 | -2.5143 | 0.013 |
| Performance-based cognitive flexibility | 0.00741 | 0.1422 | 1.007 | 0.762 | 1.331 | 0.0521 | 0.958 |
| Technostress | -0.20818 | 0.0678 | 0.812 | 0.711 | 0.927 | -3.0704 | 0.002 |
| ICT learning confidence | -0.31803 | 0.1114 | 0.728 | 0.585 | 0.905 | -2.8552 | 0.005 |
| ICT attitude | -0.12945 | 0.0808 | 0.879 | 0.750 | 1.029 | -1.6020 | 0.110 |
| Age | -0.23273 | 0.0425 | 0.792 | 0.729 | 0.861 | -5.4760 | < .001 |
| Education | 0.12875 | 0.7337 | 1.137 | 0.270 | 4.791 | 0.1755 | 0.861 |
| SES | -0.01974 | 0.4274 | 0.980 | 0.424 | 2.266 | -0.0462 | 0.963 |
| Screen time | -0.02046 | 0.1663 | 0.980 | 0.707 | 1.357 | -0.1230 | 0.902 |
| Device | 1.06277 | 0.6238 | 2.894 | 0.852 | 9.830 | 1.7037 | 0.090 |
| Motivation | 0.20658 | 0.2256 | 1.229 | 0.790 | 1.913 | 0.9156 | 0.361 |
| **GLM3 - ICT Learning confidence** |  |  |  |  |  |  |  |
| Self-reported cognitive flexibility | -0.11178 | 0.0413 | 0.894 | 0.825 | 0.970 | -2.7072 | 0.007 |
| Inattention | -0.06563 | 0.0692 | 0.936 | 0.818 | 1.072 | -0.9488 | 0.344 |
| Hyperactivity-Impulsivity | 0.05561 | 0.0802 | 1.057 | 0.903 | 1.237 | 0.6936 | 0.489 |
| Performance-based cognitive flexibility | 0.00282 | 0.0890 | 1.003 | 0.842 | 1.194 | 0.0317 | 0.975 |
| Age | -0.03663 | 0.0262 | 0.964 | 0.916 | 1.015 | -1.3983 | 0.163 |
| Education | -0.46040 | 0.4591 | 0.631 | 0.257 | 1.552 | -1.0028 | 0.317 |
| SES | -0.48965 | 0.2609 | 0.613 | 0.368 | 1.022 | -1.8770 | 0.062 |
| Screen time | -0.10203 | 0.1019 | 0.903 | 0.740 | 1.103 | -1.0012 | 0.318 |
| Device | -0.19186 | 0.3897 | 0.825 | 0.385 | 1.772 | -0.4923 | 0.623 |
| Motivation | -0.84708 | 0.1297 | 0.429 | 0.332 | 0.553 | -6.5306 | < .001 |
| **GLM4 - Technostress** |  |  |  |  |  |  |  |
| Self-reported cognitive flexibility | -0.2461 | 0.0679 | 0.782 | 0.684 | 0.893 | -3.626 | < .001 |
| Inattention | -0.0257 | 0.1137 | 0.975 | 0.780 | 1.218 | -0.226 | 0.822 |
| Hyperactivity-Impulsivity | 0.1796 | 0.1318 | 1.197 | 0.924 | 1.550 | 1.363 | 0.174 |
| Performance-based cognitive flexibility | -0.0408 | 0.1464 | 0.960 | 0.721 | 1.279 | -0.279 | 0.781 |
| Age | 0.0455 | 0.0431 | 1.047 | 0.962 | 1.139 | 1.056 | 0.292 |
| Education | -0.1791 | 0.7548 | 0.836 | 0.190 | 3.670 | -0.237 | 0.813 |
| SES | -0.6791 | 0.4289 | 0.507 | 0.219 | 1.175 | -1.583 | 0.115 |
| Screen time | 0.0446 | 0.1675 | 1.046 | 0.753 | 1.452 | 0.266 | 0.791 |
| Device | -0.8822 | 0.6408 | 0.414 | 0.118 | 1.453 | -1.377 | 0.170 |
| Motivation | -0.8171 | 0.2132 | 0.442 | 0.291 | 0.671 | -3.832 | < .001 |
