# Supplementary material for: The mediating role of ICT learning confidence and technostress between executive functions and digital skills
Source: Sci Rep. 2024 May 29;14:12343. doi: 10.1038/s41598-024-63120-w (PMC11136953; doi:10.1038/s41598-024-63120-w)
Supplement: Supplementary file 3 — Supplementary Table 2. [file 41598_2024_63120_MOESM3_ESM.docx]

| Supplementary Table 2 - Exact statistical values regarding SEM. All pathways (both direct and indirect pathways) are reported. | | | | | |  |  |  |  |  |
| --- | --- | --- | --- | --- | --- | --- | --- | --- | --- | --- |
| **Direct pathways** |  |  |  |  |  |  |  |  |  |  |
|  |  |  |  | **95% Confidence Intervals** | |  | **β 95% Confidence Intervals** | |  |  |
| **Dependent variable** | **Independent predictor** | **Estimate** | **SE** | **Lower** | **Upper** | **β** | **Lower** | **Upper** | **z** | **p** |
| Smartphone skills | Inattention | 0.02998 | 0.01032 | 0.00976 | 0.05021 | 0.180 | 0.0677 | 0.2933 | 2.91 | 0.004 |
| Smartphone skills | Hyperactivity-impulsivity | -0.03706 | 0.01445 | -0.06537 | -0.00874 | -0.197 | -0.3239 | -0.0697 | -2.57 | 0.010 |
| Smartphone skills | Technostress | -0.22613 | 0.06529 | -0.35409 | -0.09817 | -0.227 | -0.3413 | -0.1117 | -3.46 | < .001 |
| Smartphone skills | ICT learning confidence | -0.17733 | 0.06493 | -0.30459 | -0.05007 | -0.173 | -0.3005 | -0.0460 | -2.73 | 0.006 |
| Smartphone skills | Age | -0.02594 | 0.00520 | -0.03613 | -0.01575 | -0.371 | -0.4786 | -0.2634 | -4.99 | < .001 |
| Computer skills | Hyperactivity-impulsivity | -0.02225 | 0.01609 | -0.05379 | 0.00929 | -0.118 | -0.2776 | 0.0413 | -1.38 | 0.167 |
| Computer skills | Flanker | -0.00656 | 0.00202 | -0.01053 | -0.00260 | -0.189 | -0.2977 | -0.0803 | -3.24 | 0.001 |
| Computer skills | ICT learning confidence | -0.54580 | 0.07121 | -0.68538 | -0.40622 | -0.533 | -0.6329 | -0.4337 | -7.66 | < .001 |
| Computer skills | Education | 0.29308 | 0.07392 | 0.14820 | 0.43796 | 0.231 | 0.1193 | 0.3435 | 3.96 | < .001 |
| Technostress | Self-reported cognitive flexibility | -0.02862 | 0.00819 | -0.04468 | -0.01256 | -0.225 | -0.3492 | -0.1017 | -3.49 | < .001 |
| Technostress | Motivation | -0.11225 | 0.02354 | -0.15839 | -0.06612 | -0.297 | -0.4144 | -0.1800 | -4.77 | < .001 |
| ICT learning confidence | Self-reported cognitive flexibility | -0.03115 | 0.00671 | -0.04429 | -0.01800 | -0.252 | -0.3591 | -0.1439 | -4.64 | < .001 |
| ICT learning confidence | Motivation | -0.12282 | 0.02349 | -0.16886 | -0.07678 | -0.333 | -0.4523 | -0.2145 | -5.23 | < .001 |
| ICT learning confidence | SES | -0.11485 | 0.04710 | -0.20717 | -0.02252 | -0.148 | -0.2679 | -0.0284 | -2.44 | 0.015 |
| **Indirect pathways** |  |  |  |  |  |  |  |  |  |  |
| Self-reported cognitive flexibility ⇒ Technostress ⇒ Smartphone skills | | 0.006 | 0.003 | 0.002 | 0.011 | 0.051 | 0.01317 | 0.0890 | 2.575 | 0.010 |
| Self-reported cognitive flexibility ⇒ ICT learning confidence ⇒ Smartphone skills | | 0.006 | 0.002 | 0.001 | 0.010 | 0.044 | 0.00699 | 0.0802 | 2.467 | 0.014 |
| Self-reported cognitive flexibility ⇒ ICT learning confidence ⇒ Computer skills | | 0.017 | 0.004 | 0.009 | 0.025 | 0.134 | 0.07210 | 0.1962 | 4.190 | < .001 |
| Motivation ⇒ Technostress ⇒ Smartphone skills | | 0.025 | 0.009 | 0.007 | 0.044 | 0.067 | 0.02381 | 0.1108 | 2.726 | 0.006 |
| Motivation ⇒ ICT learning confidence ⇒ Smartphone skills | | 0.022 | 0.010 | 0.003 | 0.041 | 0.058 | 0.00799 | 0.1075 | 2.261 | 0.024 |
| Motivation ⇒ CARS_total ⇒ Computer skills | | 0.067 | 0.016 | 0.035 | 0.099 | 0.178 | 0.10247 | 0.2531 | 4.073 | < .001 |
| SES ⇒ ICT learning confidence ⇒ Smartphone skills | | 0.020 | 0.011 | -0.001 | 0.042 | 0.026 | -0.00222 | 0.0536 | 1.866 | 0.062 |
| SES ⇒ ICT learning confidence⇒ Computer skills | | 0.063 | 0.026 | 0.011 | 0.114 | 0.079 | 0.01397 | 0.1440 | 2.374 | 0.018 |
