# Supplementary material for: The mediating role of ICT learning confidence and technostress between executive functions and digital skills
Source: Sci Rep. 2024 May 29;14:12343. doi: 10.1038/s41598-024-63120-w (PMC11136953; doi:10.1038/s41598-024-63120-w)
Supplement: Supplementary file 4 — Supplementary Table 3. [file 41598_2024_63120_MOESM4_ESM.docx]

**Supplementary Table 3.** – Correlational coefficients across all included variables.

|  | 1. | 2. | 3. | 4. | 5. | 6. | 7. | 8. | 9. | 10. | 11. | 12. | 13. | 14. | 15. | 16. |
| --- | --- | --- | --- | --- | --- | --- | --- | --- | --- | --- | --- | --- | --- | --- | --- | --- |
| 1. Self-reported cognitive flexibility | - |  |  |  |  |  |  |  |  |  |  |  |  |  |  |  |
| 2. Inattention | .065 | - |  |  |  |  |  |  |  |  |  |  |  |  |  |  |
| 3. Hyperactivity-impulsivity | .035 | .643*** | - |  |  |  |  |  |  |  |  |  |  |  |  |  |
| 4. Performance-based cognitive flexibility | -.019 | .083 | .092 | - |  |  |  |  |  |  |  |  |  |  |  |  |
| 5. Performance-based cognitive control | .080 | -.071 | -.064 | -.036 | - |  |  |  |  |  |  |  |  |  |  |  |
| 6. Technostress | -.278*** | .054 | .112 | .047 | -.116 | - |  |  |  |  |  |  |  |  |  |  |
| 7. ICT Confidence scale | -.256*** | -.080 | -.005 | .058 | -.002 | .482*** | - |  |  |  |  |  |  |  |  |  |
| 8. ICT Attitude | -.007 | -.071 | -.029 | -.009 | .074 | .052 | .018 | - |  |  |  |  |  |  |  |  |
| 9. Age | .153* | .109 | .132* | .041 | .074 | .117 | -.012 | .058 | - |  |  |  |  |  |  |  |
| 10. Education | .181** | .060 | .074 | -.010 | .123* | -.036 | -.115 | -.019 | .402*** | - |  |  |  |  |  |  |
| 11. SES | .097 | .010 | .033 | -.057 | .085 | -.113 | -.145* | -.192** | .180** | .259*** | - |  |  |  |  |  |
| 12. Device | .120* | .046 | .009 | .059 | .096 | -.119 | -.086 | -.033 | .046 | .227*** | .124* | - |  |  |  |  |
| 13. Screen time | .011 | .164** | .261*** | .019 | .050 | .036 | -.036 | .217*** | -.017 | -.105 | -.132* | .079 | - |  |  |  |
| 14. Motivation | .111 | -.002 | -.078 | -.111 | .121* | -.291 | -384 | .050 | -.382*** | -.120* | -.002 | .002 | -.002 | - |  |  |
| 15. Smartphone skills | .138* | .065 | -.059 | -.038 | -.038 | -.346*** | -.382*** | -.126* | -.241*** | -.118 | .083 | .110 | -.033 | .211*** | - |  |
| 16. Computer skills | .300*** | .047 | -.025 | .023 | -.112 | -.281*** | -.405*** | -.011 | .250*** | .262*** | .140* | .139* | .011 | .107 | .415*** | - |

*Note.* * p < .05, ** p < .01, *** p < .001
